# Supplementary figures and images for: Interplay of human macrophage response and natural resistance of infection by L. (V.) panamensis to pentavalent antimony
Source: PLoS Negl Trop Dis. 2025 Oct 6;19(10):e0013600. doi: 10.1371/journal.pntd.0013600 (PMC12517518; doi:10.1371/journal.pntd.0013600)

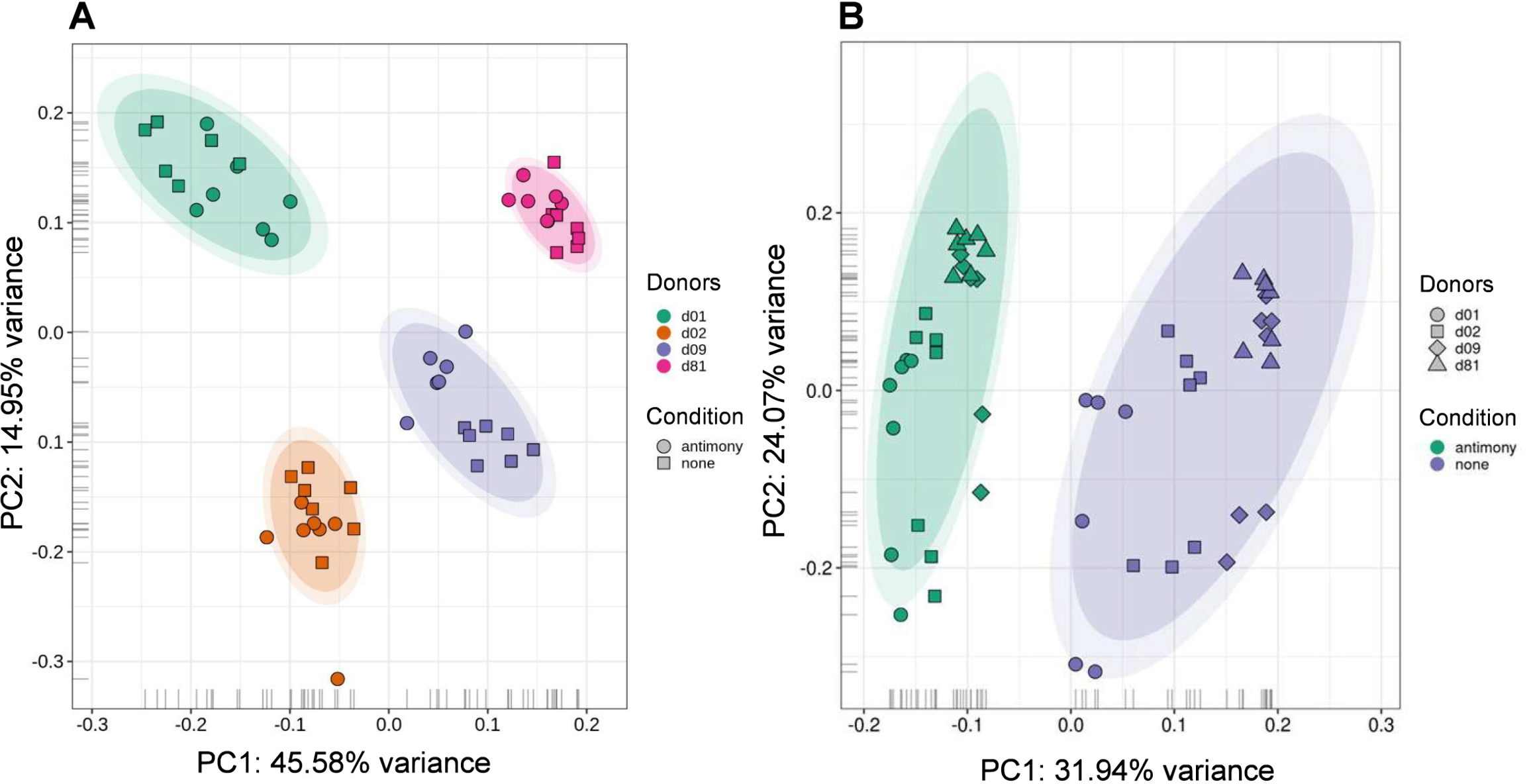

Supplement: S1 Fig — (V.) panamensis in absence or presence of antimony. Principal component analysis (PCA) plot analysis of (A) normalized and SVA-adjusted (for donor) RNA-seq expression values of macrophage samples from 4 donors, uninfected (none) or infected with strains of zym 2.2 and zym 2.3, in the absence or presence of 32 µg SbV/mL, or (B) same analysis without SVA adjustment. Results (including a variance partition plot available on our complete log of analyses at https://doi.org/10.5281/zenodo.16944615) suggest that the majority of the attributable variance is associated with donor over any other factor. In contrast, when PCA was performed with donor as the primary factor, the results indicated that drug treatment was the dominant factor in the data, followed by infecting zymodeme. (TIF) [file pntd.0013600.s001.tif]

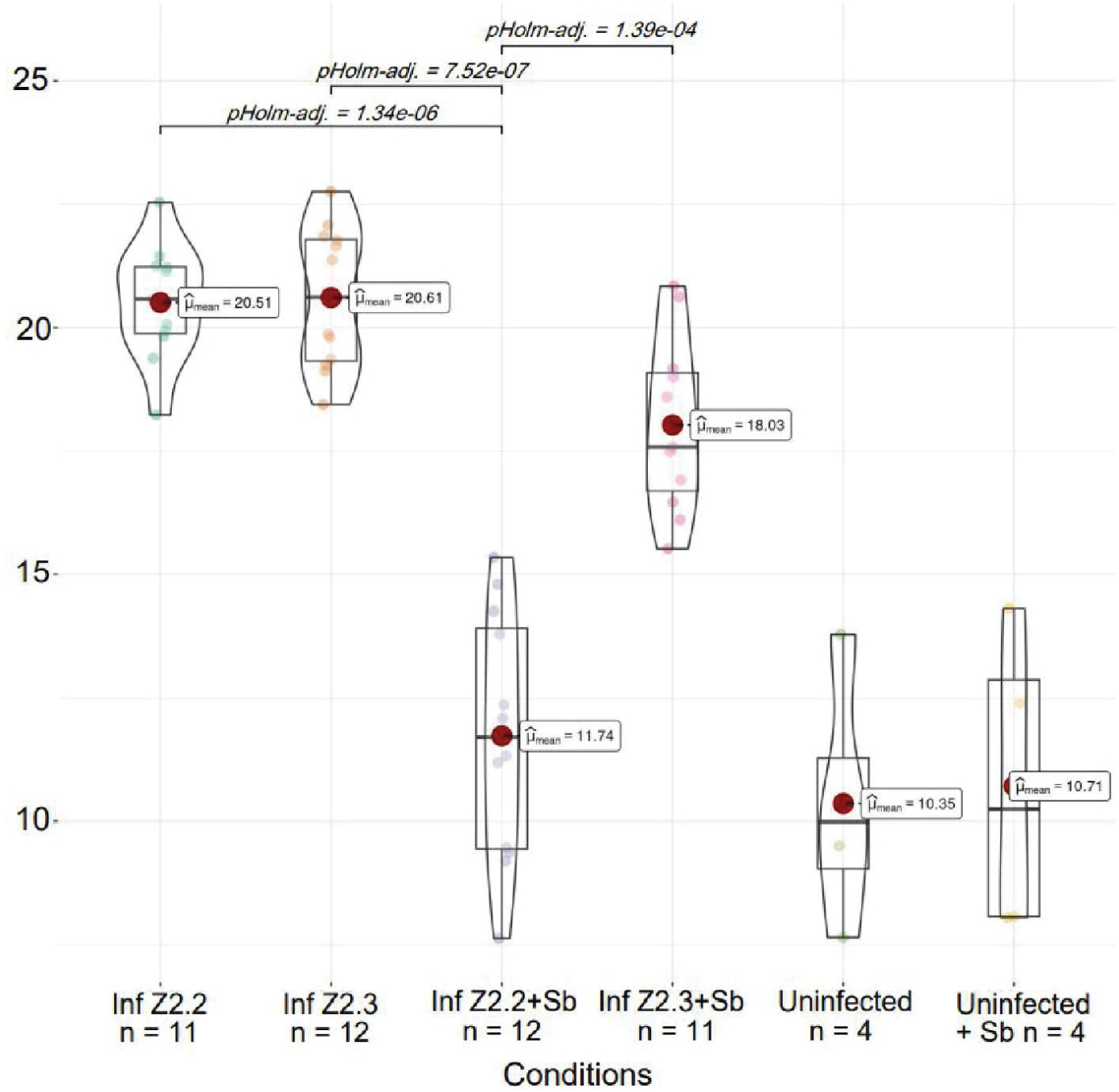

Supplement: S2 Fig — (V.) panamensis zymodemes 2.2 and 2.3, exposed or unexposed to antimony treatment. The number of reads mapped to the parasite was observed in every sample, collected by infection status, and plotted on the log2 scale. The small numbers of reads observed in the uninfected samples shows the low rate of spurious mapping to highly conserved regions; which is similar to the number of reads observed in the drug-treated zymodeme 2.2 samples. The drug-treated zymodeme 2.3 samples retained a significantly higher number of parasite reads, while the untreated samples have the most reads and are indistinguishable from each other. Each red dot describes the mean number of reads observed in that sample group. The box defines the inner-quartile range. The whiskers delineate the outer quartiles. Each colored dot within the violin plot describes the mapping rate of that sample; the density of samples defines the shape of the violin. The degree of significance was defined by a Bonferroni-Holm adjusted P value. (TIF) [file pntd.0013600.s002.tif]

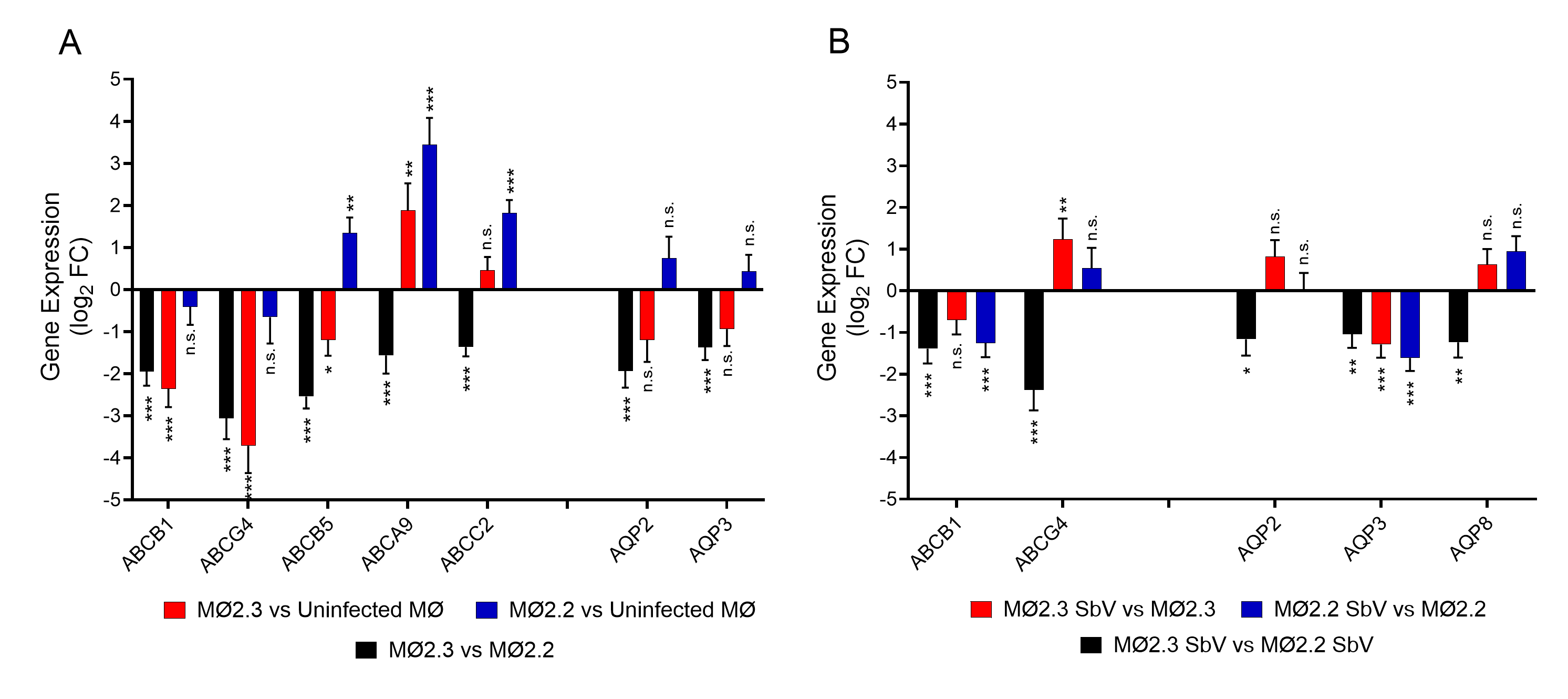

Supplement: S3 Fig — (V.) panamensis. (A) Differential gene expression between macrophages infected with 2.3 and 2.2 strains in the absence of drug. (B) Differential gene expression between macrophages infected with 2.3 and 2.2 strains in the presence of antimony. Data corresponds to transcriptome analysis of monocyte-derived macrophages from four healthy donors. adj. P < 0.05. * P < 0.05 ** P ≤ 0.01 ***. (TIF) [file pntd.0013600.s003.tif]
