# Supplementary material for: Interplay of human macrophage response and natural resistance of infection by L. (V.) panamensis to pentavalent antimony
Source: PLoS Negl Trop Dis. 2025 Oct 6;19(10):e0013600. doi: 10.1371/journal.pntd.0013600 (PMC12517518; doi:10.1371/journal.pntd.0013600)
Supplement: S3 Table — (V.) panamensis strains of zymodemes 2.2 and 2.3 in the absence of drug. (DOCX) [file pntd.0013600.s006.docx]

**S3 Table.** Top 10 most enriched pathways among up- and downregulated genes in primary human macrophages infected with *L. (V.) panamensis* strains of zymodemes 2.2 and 2.3 in the absence of drug

| **Top 10 significant pathways** | | |
| --- | --- | --- |
| **Infection 2.3 vs Uninfected (Genes Upregulated)** | | |
| ***Reactome Pathways*** | *Recall* | *P value* |
| Immune System | 0,040 | 0,031 |
| Cytokine Signaling in Immune system | 0,102 | 0,000 |
| Peptide ligand-binding receptors | 0,046 | 0,006 |
| Interferon Signaling | 0,171 | 0,000 |
| Interferon alpha/beta signaling | 0,282 | 0,000 |
| Antiviral mechanism by IFN-stimulated genes | 0,141 | 0,000 |
| Chemokine receptors bind chemokines | 0,175 | 0,000 |
| OAS antiviral response | 0,556 | 0,000 |
| Interferon gamma signaling | 0,161 | 0,000 |
| Interleukin-10 signaling | 0,222 | 0,000 |
|  |  |  |
| ***Gene Ontology (Biological Process)*** |  |  |
| positive regulation of pyroptosis | 0,800 | 0,002 |
| tryptophan catabolic process to kynurenine | 0,800 | 0,002 |
| interleukin-27-mediated signaling pathway | 0,714 | 0,000 |
| cytolysis in another organism | 0,667 | 0,006 |
| DNA cytosine deamination | 0,556 | 0,001 |
| tryptophan catabolic process | 0,556 | 0,001 |
| indole-containing compound catabolic process | 0,556 | 0,001 |
| ovarian cumulus expansion | 0,500 | 0,020 |
| cytolysis | 0,500 | 0,025 |
| positive regulation of natural killer cell chemotaxis | 0,500 | 0,039 |
|  |  |  |
| ***Gene Ontology (Molecular Function)*** |  |  |
| 2'-5'-oligoadenylate synthetase activity | 1,000 | 0,000 |
| tryptophan 2,3-dioxygenase activity | 1,000 | 0,000 |
| indoleamine 2,3-dioxygenase activity | 1,000 | 0,002 |
| CCR1 chemokine receptor binding | 0,667 | 0,000 |
| exoribonuclease II activity | 0,667 | 0,025 |
| CCR5 chemokine receptor binding | 0,571 | 0,000 |
| deoxycytidine deaminase activity | 0,556 | 0,000 |
| cytidine deaminase activity | 0,417 | 0,001 |
| CXCR chemokine receptor binding | 0,278 | 0,016 |
| pattern recognition receptor activity | 0,265 | 0,000 |
|  |  |  |
| **Infection 2.2 vs Uninfected (Genes Upregulated)** | | |
| ***Reactome Pathways*** | *Recall* | *P value* |
| Constitutive Signaling by NOTCH1 t(7;9)(NOTCH1:M1580_K2555) Translocation Mutant | 0,714 | 0,000 |
| Signaling by NOTCH1 t(7;9)(NOTCH1:M1580_K2555) Translocation Mutant | 0,714 | 0,000 |
| Constitutive Signaling by NOTCH1 HD Domain Mutants | 0,333 | 0,030 |
| Signaling by NOTCH1 HD Domain Mutants in Cancer | 0,333 | 0,030 |
|  |  |  |
| ***Gene Ontology (Biological Process)*** |  |  |
| negative regulation of inner ear receptor cell differentiation | 0,750 | 0,017 |
| negative regulation of inner ear auditory receptor cell differentiation | 0,750 | 0,017 |
| negative regulation of mechanoreceptor differentiation | 0,750 | 0,017 |
| inhibition of neuroepithelial cell differentiation | 0,667 | 0,002 |
| xenobiotic transport across blood-brain barrier | 0,500 | 0,043 |
| regulation of forebrain neuron differentiation | 0,400 | 0,020 |
| vascular associated smooth muscle cell development | 0,333 | 0,025 |
| pulmonary valve morphogenesis | 0,316 | 0,001 |
| dorsal aorta development | 0,300 | 0,007 |
| pulmonary valve development | 0,261 | 0,003 |
|  |  |  |
| ***Gene Ontology (Molecular Function)*** |  |  |
| toxin transmembrane transporter activity | 0,375 | 0,047 |
| ankyrin binding | 0,263 | 0,001 |
| Notch binding | 0,192 | 0,032 |
| voltage-gated channel activity | 0,065 | 0,035 |
| monoatomic ion channel activity | 0,047 | 0,011 |
| DNA-binding transcription activator activity, RNA polymerase II-specific | 0,047 | 0,022 |
| DNA-binding transcription activator activity | 0,046 | 0,027 |
| metal ion transmembrane transporter activity | 0,045 | 0,034 |
|  |  |  |
| **Infection 2.3 vs 2.2 (Genes Upregulated)** | | |
| ***Reactome Pathways*** | *Recall* | *P value* |
| OAS antiviral response | 0,556 | 0,000 |
| Interferon alpha/beta signaling | 0,268 | 0,000 |
| Negative regulators of DDX58/IFIH1 signaling | 0,229 | 0,004 |
| Interleukin-10 signaling | 0,200 | 0,003 |
| Chemokine receptors bind chemokines | 0,175 | 0,000 |
| Interferon gamma signaling | 0,161 | 0,000 |
| Interferon Signaling | 0,161 | 0,000 |
| Antiviral mechanism by IFN-stimulated genes | 0,141 | 0,000 |
| ISG15 antiviral mechanism | 0,129 | 0,004 |
| Peptide ligand-binding receptors | 0,067 | 0,019 |
|  |  |  |
| ***Gene Ontology (Biological Process)*** |  |  |
| positive regulation of pyroptosis | 0,800 | 0,000 |
| interleukin-27-mediated signaling pathway | 0,714 | 0,000 |
| cytolysis in another organism | 0,667 | 0,001 |
| regulation of type III interferon production | 0,600 | 0,023 |
| type III interferon production | 0,600 | 0,023 |
| cytolysis | 0,500 | 0,006 |
| leukocyte chemotaxis involved in inflammatory response | 0,500 | 0,007 |
| positive regulation of chronic inflammatory response | 0,500 | 0,012 |
| ovarian cumulus expansion | 0,500 | 0,024 |
| positive regulation of natural killer cell chemotaxis | 0,500 | 0,025 |
|  |  |  |
| ***Gene Ontology (Molecular Function)*** |  |  |
| 2'-5'-oligoadenylate synthetase activity | 1,000 | 0,000 |
| CCR1 chemokine receptor binding | 0,667 | 0,000 |
| exoribonuclease II activity | 0,667 | 0,025 |
| CCR5 chemokine receptor binding | 0,571 | 0,000 |
| deoxycytidine deaminase activity | 0,444 | 0,004 |
| CXCR chemokine receptor binding | 0,333 | 0,000 |
| cytidine deaminase activity | 0,333 | 0,014 |
| chemokine activity | 0,300 | 0,000 |
| pattern recognition receptor activity | 0,235 | 0,000 |
| chemokine receptor binding | 0,208 | 0,000 |
|  |  |  |
| **Infection 2.3 vs Uninfected (Genes Downregulated)** | | |
| ***Reactome Pathways*** |  |  |
| There was no enrichment |  |  |
|  |  |  |
| ***Gene Ontology (Biological Process)*** |  |  |
| regulation of glomerular filtration | 0,444 | 0,001 |
| renal system process involved in regulation of blood volume | 0,286 | 0,011 |
| renal system process involved in regulation of systemic arterial blood pressure | 0,208 | 0,008 |
| carboxylic acid transport | 0,042 | 0,023 |
| organic acid transport | 0,042 | 0,023 |
| positive regulation of intracellular signal transduction | 0,031 | 0,004 |
| response to insulin | 0,030 | 0,014 |
| positive regulation of signal transduction | 0,027 | 0,003 |
| positive regulation of cell communication | 0,025 | 0,016 |
| positive regulation of multicellular organismal process | 0,024 | 0,046 |
|  |  |  |
| ***Gene Ontology (Molecular Function)*** |  |  |
| bile acid binding | 0,300 | 0,033 |
| immunoglobulin receptor activity | 0,300 | 0,047 |
| ABC-type xenobiotic transporter activity | 0,214 | 0,006 |
| phosphate ion transmembrane transporter activity | 0,200 | 0,043 |
| xenobiotic transmembrane transporter activity | 0,135 | 0,026 |
| cargo receptor activity | 0,080 | 0,037 |
| molecular transducer activity | 0,026 | 0,011 |
| signaling receptor activity | 0,026 | 0,011 |
|  |  |  |
| **Infection 2.2 vs Uninfected** | | |
| No data |  |  |
|  |  |  |
| **Infection 2.3 vs 2.2 (Genes Downregulated)** | | |
| ***Reactome Pathways*** |  |  |
| Neuronal System | ND | 0,005 |
| Constitutive Signaling by NOTCH1 t(7;9)(NOTCH1 M1580 K2555) Translocation Mutant | ND | 0,005 |
| Signaling by NOTCH1 t(7;9)(NOTCH1 M1580 K2555) Translocation Mutant | ND | 0,005 |
| Constitutive Signaling by NOTCH1 HD Domain Mutants | ND | 0,006 |
| Signaling by NOTCH1 HD Domain Mutants in Cancer | ND | 0,006 |
| Abacavir ADME | ND | 0,008 |
| Activated NOTCH1 Transmits Signal to the Nucleus | ND | 0,018 |
| Abacavir Transmembrane Transport | ND | 0,018 |
| Nuclear Events (Kinase and Transcription Factor Activation) | ND | 0,018 |
| Activation of the Pre-Replicative Complex | ND | 0,018 |
|  |  |  |
| ***Gene Ontology (Biological Process)*** |  |  |
| response to interleukin-8 | 1,000 | 0,030 |
| cellular response to interleukin-8 | 1,000 | 0,030 |
| xenobiotic transport across blood-brain barrier | 0,667 | 0,013 |
| positive regulation of skeletal muscle cell differentiation | 0,667 | 0,014 |
| regulation of fibrinolysis | 0,278 | 0,038 |
| positive regulation of cardiac muscle tissue growth | 0,207 | 0,030 |
| positive regulation of interleukin-2 production | 0,200 | 0,015 |
| negative regulation of blood vessel endothelial cell migration | 0,176 | 0,012 |
| cell migration involved in sprouting angiogenesis | 0,164 | 0,017 |
| regulation of dendritic spine development | 0,153 | 0,013 |
|  |  |  |
| ***Gene Ontology (Molecular Function)*** |  |  |
| minus-end-directed microtubule motor activity | 0,294 | 0,022 |
| dynein light intermediate chain binding | 0,214 | 0,043 |
| phosphatidylserine binding | 0,133 | 0,024 |
| serine-type endopeptidase inhibitor activity | 0,107 | 0,038 |
| transporter activity | 0,045 | 0,033 |
