# Supplementary material for: Interplay of human macrophage response and natural resistance of infection by L. (V.) panamensis to pentavalent antimony
Source: PLoS Negl Trop Dis. 2025 Oct 6;19(10):e0013600. doi: 10.1371/journal.pntd.0013600 (PMC12517518; doi:10.1371/journal.pntd.0013600)
Supplement: S4 Table — (V.) panamensis strains of zymodemes 2.2 and 2.3 in the presence of antimony. (DOCX) [file pntd.0013600.s007.docx]

**S4 Table.** Top 10 most enriched pathways among up- and downregulated genes in primary human macrophages infected with *L. (V.) panamensis* strains of zymodemes 2.2 and 2.3 in the presence of antimony.

| **Top 10 significant pathways** | | |
| --- | --- | --- |
| **Infection 2.3 Sb vs 2.3 (Genes Upregulated)** | | |
| ***Reactome Pathways*** | *Recall* | *P value* |
| Metallothioneins bind metals | 0,545 | 0,000 |
| Response to metal ions | 0,429 | 0,000 |
| Synthesis of bile acids and bile salts via 24-hydroxycholesterol | 0,286 | 0,000 |
| Synthesis of bile acids and bile salts via 27-hydroxycholesterol | 0,267 | 0,000 |
| Synthesis of bile acids and bile salts via 7alpha-hydroxycholesterol | 0,217 | 0,000 |
| Packaging Of Telomere Ends | 0,216 | 0,000 |
| RNA Polymerase I Promoter Opening | 0,210 | 0,000 |
| Recognition and association of DNA glycosylase with site containing an affected purine | 0,204 | 0,000 |
| Depurination | 0,204 | 0,000 |
| Cleavage of the damaged purine | 0,204 | 0,000 |
|  |  |  |
| ***Gene Ontology (Biological Process)*** |  |  |
| cellular response to jasmonic acid stimulus | 1,000 | 0,000 |
| response to jasmonic acid | 1,000 | 0,000 |
| muscle hyperplasia | 1,000 | 0,024 |
| smooth muscle hyperplasia | 1,000 | 0,024 |
| cellular response to iron ion | 0,571 | 0,045 |
| daunorubicin metabolic process | 0,444 | 0,000 |
| stress response to copper ion | 0,429 | 0,000 |
| detoxification of copper ion | 0,429 | 0,000 |
| polyketide metabolic process | 0,400 | 0,000 |
| aminoglycoside antibiotic metabolic process | 0,400 | 0,000 |
|  |  |  |
| ***Gene Ontology (Molecular Function)*** |  |  |
| phenanthrene 9,10-monooxygenase activity | 1,000 | 0,000 |
| ketosteroid monooxygenase activity | 0,800 | 0,000 |
| dihydrotestosterone 17-beta-dehydrogenase activity | 0,750 | 0,000 |
| trans-1,2-dihydrobenzene-1,2-diol dehydrogenase activity | 0,667 | 0,006 |
| indanol dehydrogenase activity | 0,667 | 0,006 |
| androsterone dehydrogenase activity | 0,500 | 0,000 |
| 5alpha-androstane-3beta,17beta-diol dehydrogenase activity | 0,500 | 0,000 |
| androstan-3-alpha,17-beta-diol dehydrogenase activity | 0,444 | 0,000 |
| bile acid binding | 0,400 | 0,000 |
| alditol:NADP+ 1-oxidoreductase activity | 0,333 | 0,000 |
|  |  |  |
| **Infection 2.2 Sb vs 2.2 (Genes Upregulated)** | | |
| ***Reactome Pathways*** | *Recall* | *P value* |
| Metallothioneins bind metals | 0,455 | 0,000 |
| Response to metal ions | 0,357 | 0,002 |
| Synthesis of bile acids and bile salts via 24-hydroxycholesterol | 0,286 | 0,001 |
| Packaging Of Telomere Ends | 0,275 | 0,000 |
| Synthesis of bile acids and bile salts via 27-hydroxycholesterol | 0,267 | 0,002 |
| Recognition and association of DNA glycosylase with site containing an affected purine | 0,259 | 0,000 |
| Cleavage of the damaged purine | 0,259 | 0,000 |
| Depurination | 0,259 | 0,000 |
| RNA Polymerase I Promoter Opening | 0,258 | 0,000 |
| DNA methylation | 0,250 | 0,000 |
|  |  |  |
| ***Gene Ontology (Biological Process)*** |  |  |
| response to jasmonic acid | 1,000 | 0,000 |
| cellular response to jasmonic acid stimulus | 1,000 | 0,000 |
| muscle hyperplasia | 1,000 | 0,002 |
| smooth muscle hyperplasia | 1,000 | 0,002 |
| smooth muscle adaptation | 0,500 | 0,014 |
| daunorubicin metabolic process | 0,444 | 0,000 |
| aminoglycoside antibiotic metabolic process | 0,400 | 0,000 |
| doxorubicin metabolic process | 0,400 | 0,000 |
| polyketide metabolic process | 0,400 | 0,000 |
| detoxification of copper ion | 0,357 | 0,000 |
|  |  |  |
| ***Gene Ontology (Molecular Function)*** |  |  |
| phenanthrene 9,10-monooxygenase activity | 1,000 | 0,000 |
| ketosteroid monooxygenase activity | 0,800 | 0,000 |
| dihydrotestosterone 17-beta-dehydrogenase activity | 0,750 | 0,000 |
| indanol dehydrogenase activity | 0,667 | 0,026 |
| trans-1,2-dihydrobenzene-1,2-diol dehydrogenase activity | 0,667 | 0,026 |
| androsterone dehydrogenase activity | 0,500 | 0,000 |
| 5alpha-androstane-3beta,17beta-diol dehydrogenase activity | 0,500 | 0,000 |
| androstan-3-alpha,17-beta-diol dehydrogenase activity | 0,444 | 0,000 |
| bile acid binding | 0,400 | 0,000 |
| alditol:NADP+ 1-oxidoreductase activity | 0,333 | 0,000 |
|  |  |  |
| **Infection 2.3 Sb vs 2.2 Sb (Genes Upregulated)** | | |
| ***Reactome Pathways*** | *Recall* | *P value* |
| OAS antiviral response | 0,556 | 0,000 |
| Interferon alpha/beta signaling | 0,324 | 0,000 |
| Packaging Of Telomere Ends | 0,176 | 0,007 |
| Negative regulators of DDX58/IFIH1 signaling | 0,171 | 0,009 |
| Interferon Signaling | 0,171 | 0,000 |
| Depurination | 0,167 | 0,012 |
| Recognition and association of DNA glycosylase with site containing an affected purine | 0,167 | 0,012 |
| Cleavage of the damaged purine | 0,167 | 0,012 |
| Interferon gamma signaling | 0,161 | 0,000 |
| Chemokine receptors bind chemokines | 0,158 | 0,003 |
|  |  |  |
| ***Gene Ontology (Biological Process)*** |  |  |
| interleukin-27-mediated signaling pathway | 0,714 | 0,000 |
| negative regulation of IP-10 production | 0,667 | 0,038 |
| type III interferon production | 0,600 | 0,028 |
| regulation of type III interferon production | 0,600 | 0,028 |
| ISG15-protein conjugation | 0,500 | 0,026 |
| MDA-5 signaling pathway | 0,455 | 0,000 |
| regulation of chronic inflammatory response | 0,400 | 0,011 |
| negative regulation of viral genome replication | 0,364 | 0,000 |
| regulation of T-helper 1 cell differentiation | 0,364 | 0,015 |
| response to interferon-beta | 0,353 | 0,000 |
|  |  |  |
| ***Gene Ontology (Molecular Function)*** |  |  |
| 2'-5'-oligoadenylate synthetase activity | 1,000 | 0,000 |
| interleukin-27 receptor binding | 1,000 | 0,018 |
| exoribonuclease II activity | 0,667 | 0,008 |
| death receptor activity | 0,231 | 0,046 |
| CXCR chemokine receptor binding | 0,222 | 0,035 |
| pattern recognition receptor activity | 0,206 | 0,000 |
| chemokine activity | 0,180 | 0,000 |
| double-stranded RNA binding | 0,164 | 0,000 |
| tumor necrosis factor receptor superfamily binding | 0,143 | 0,006 |
| hydrolase activity, acting on carbon-nitrogen (but not peptide) bonds, in cyclic amidines | 0,139 | 0,043 |
|  |  |  |
| **Infection 2.3 Sb vs 2.3 (Genes Downregulated)** | | |
| ***Reactome Pathways*** | *Recall* | *P value* |
| Defective B3GALT6 causes EDSP2 and SEMDJL1 | 0,400 | 0,013 |
| Defective B3GAT3 causes JDSSDHD | 0,400 | 0,013 |
| Defective B4GALT7 causes EDS, progeroid type | 0,400 | 0,013 |
| Cell-extracellular matrix interactions | 0,333 | 0,002 |
| Interleukin-10 signaling | 0,289 | 0,009 |
| Chondroitin sulfate/dermatan sulfate metabolism | 0,245 | 0,040 |
| Maturation of protein 3a | 0,222 | 0,043 |
| Maturation of protein 3a | 0,222 | 0,043 |
| Phosphorylation of CD3 and TCR zeta chains | 0,217 | 0,017 |
| Glycosaminoglycan metabolism | 0,154 | 0,002 |
|  |  |  |
| ***Gene Ontology (Biological Process)*** |  |  |
| positive regulation of metanephric mesenchymal cell migration by platelet-derived growth factor receptor-beta signaling pathway | 1,000 | 0,020 |
| positive regulation of metanephric mesenchymal cell migration | 1,000 | 0,020 |
| regulation of metanephric mesenchymal cell migration by platelet-derived growth factor receptor-beta signaling pathway | 1,000 | 0,020 |
| regulation of ovarian follicle development | 0,667 | 0,006 |
| cell migration involved in kidney development | 0,667 | 0,006 |
| fatty-acyl-CoA catabolic process | 0,500 | 0,006 |
| regulation of eosinophil migration | 0,500 | 0,039 |
| T cell activation via T cell receptor contact with antigen bound to MHC molecule on antigen presenting cell | 0,444 | 0,021 |
| negative regulation of lymphocyte migration | 0,357 | 0,017 |
| eosinophil migration | 0,355 | 0,000 |
|  |  |  |
| ***Gene Ontology (Molecular Function)*** |  |  |
| profilin binding | 0,500 | 0,002 |
| platelet-derived growth factor binding | 0,455 | 0,008 |
| platelet-derived growth factor receptor binding | 0,357 | 0,033 |
| collagen binding | 0,188 | 0,001 |
| protein tyrosine phosphatase activity | 0,140 | 0,009 |
| sialyltransferase activity | 0,136 | 0,001 |
| growth factor binding | 0,127 | 0,002 |
| integrin binding | 0,120 | 0,002 |
| extracellular matrix structural constituent | 0,108 | 0,019 |
| actin binding | 0,088 | 0,000 |
|  |  |  |
| **Infection 2.2 Sb vs 2.2 (Genes Downregulated)** | | |
| ***Reactome Pathways*** | *Recall* | *P value* |
| Attachment and Entry | 0,421 | 0,032 |
| Defective B3GALT6 causes EDSP2 and SEMDJL1 | 0,400 | 0,050 |
| Defective B3GAT3 causes JDSSDHD | 0,400 | 0,050 |
| Defective B4GALT7 causes EDS, progeroid type | 0,400 | 0,050 |
| Cell recruitment (pro-inflammatory response) | 0,240 | 0,030 |
| Purinergic signaling in leishmaniasis infection | 0,240 | 0,030 |
| Inflammasomes | 0,200 | 0,041 |
| Extracellular matrix organization | 0,158 | 0,007 |
|  |  |  |
| ***Gene Ontology (Biological Process)*** |  |  |
| negative regulation of low-density lipoprotein particle receptor catabolic process | 1,000 | 0,047 |
| natural killer cell chemotaxis | 0,500 | 0,016 |
| positive regulation of DNA-templated DNA replication | 0,429 | 0,022 |
| negative regulation of bone remodeling | 0,368 | 0,000 |
| negative regulation of bone resorption | 0,353 | 0,003 |
| negative regulation of tissue remodeling | 0,304 | 0,001 |
| eosinophil chemotaxis | 0,296 | 0,003 |
| eosinophil migration | 0,290 | 0,001 |
| cyclic purine nucleotide metabolic process | 0,268 | 0,006 |
| cyclic nucleotide metabolic process | 0,262 | 0,008 |
|  |  |  |
| ***Gene Ontology (Molecular Function)*** |  |  |
| profilin binding | 0,500 | 0,010 |
| death receptor activity | 0,462 | 0,008 |
| ankyrin binding | 0,368 | 0,013 |
| cyclase activity | 0,333 | 0,019 |
| phosphorus-oxygen lyase activity | 0,292 | 0,050 |
| collagen binding | 0,217 | 0,001 |
| growth factor binding | 0,157 | 0,002 |
| potassium channel activity | 0,137 | 0,042 |
| integrin binding | 0,133 | 0,000 |
| calmodulin binding | 0,131 | 0,001 |
|  |  |  |
| **Infection 2.3 Sb vs 2.2 Sb (Genes Downregulated)** | | |
| ***Reactome Pathways*** | *Recall* | *P value* |
| Passive transport by Aquaporins | 0,231 | 0,033 |
| Vasopressin regulates renal water homeostasis via Aquaporins | 0,116 | 0,025 |
| Aquaporin-mediated transport | 0,115 | 0,005 |
|  |  |  |
| ***Gene Ontology (Biological Process)*** |  |  |
| positive regulation of immune system process | 0,028 | 0,041 |
| regulation of multicellular organismal development | 0,027 | 0,003 |
| monoatomic ion transmembrane transport | 0,025 | 0,042 |
| positive regulation of response to stimulus | 0,022 | 0,009 |
| transmembrane transport | 0,022 | 0,012 |
| regulation of multicellular organismal process | 0,021 | 0,000 |
| cell surface receptor signaling pathway | 0,019 | 0,034 |
| regulation of response to stimulus | 0,018 | 0,005 |
| transport | 0,017 | 0,008 |
| multicellular organism development | 0,017 | 0,041 |
|  |  |  |
| ***Gene Ontology (Molecular Function)*** |  |  |
| interleukin-8 binding | 0,667 | 0,015 |
| water channel activity | 0,188 | 0,011 |
| water transmembrane transporter activity | 0,158 | 0,018 |
| cytoskeletal motor activity | 0,070 | 0,005 |
| monoatomic cation channel activity | 0,039 | 0,016 |
| channel activity | 0,036 | 0,002 |
| passive transmembrane transporter activity | 0,035 | 0,002 |
| transmembrane transporter activity | 0,028 | 0,005 |
| transporter activity | 0,027 | 0,005 |
